# Disability and dignity: a qualitative systematic review of WASH-related barriers, facilitators and interventions for adults with musculoskeletal disabilities

**DOI:** 10.3389/fpubh.2026.1799013

**Published:** 2026-08-21

**Authors:** Soumyaranjan Sahu, Manisha Jnyanajyoti, Jaya Singh Kshatri, Subrata Kumar Palo, Sanghamitra Pati

**Affiliations:** ICMR-Regional Medical Research Center, Bhubaneswar, India

**Keywords:** barriers and facilitators, inclusive sanitation, musculoskeletal disabilities, systematic review, WASH access

## Abstract

**Background:**

Adults with musculoskeletal disabilities (MSDs) including arthritis, spinal cord injuries, amputations, and congenital deformities face significant barriers in accessing water, sanitation, and hygiene (WASH) services. These barriers compromise health, dignity, and independence, particularly in low- and middle-income countries. This systematic review aimed to identify WASH-related barriers and facilitators for adults with MSDs and synthesize existing interventions to promote inclusive WASH access.

**Methods:**

This systematic review was conducted following PRISMA 2020 guidelines. We searched MEDLINE, Embase, and CENTRAL databases up to March 1, 2025, using MeSH and free-text terms. Inclusion criteria were primary research studies involving adults (≥18 years) with MSDs, reporting on WASH barriers, facilitators, or interventions. Only qualitative or mixed-methods studies published in English were included. Studies involving children, lacking primary data, or behind paywalls were excluded. Two independent reviewers conducted screening and full-text appraisal using Covidence. Data were extracted using a standardized template, and findings were thematically synthesized. Study quality was assessed using the NIH Quality Assessment Tool.

**Results:**

From 5,301 initial records, 19 studies met the inclusion criteria, representing diverse geographical settings. Key barriers included inaccessible infrastructure (e.g., lack of ramps or handrails), stigma, poverty, exclusion from planning, and inadequate menstrual hygiene management. Facilitators included home modifications, affordable technology (e.g., biosand filters), caregiver and community support, gender-sensitive WASH education, and inclusive policies. Interventions ranged from inclusive Community-Led Total Sanitation (CLTS) programs to disability-aware infrastructure designs. Despite progress, participation of adults with MSDs in WASH planning and services remained limited.

**Conclusion:**

This review highlights persistent exclusion of adults with MSDs from WASH access. Inclusive design, community engagement, and policy-level integration are essential for achieving equitable WASH services. Future research should evaluate the scalability and effectiveness of low-cost interventions, particularly in resource-constrained settings. However, the findings should be interpreted considering the absence of meta-analysis and formal risk-of-bias assessment.

## 1 Introduction

The Sustainable Development Goals (SDG) 6.2 aims to eliminate open defecation and ensure access to improved sanitation for all by 2030, emphasizing support for vulnerable groups, including people with disabilities (1). Ensuring disability-inclusive WASH services also contributes to SDG 3 (Good Health and Well-being) by reducing health risks, and SDG 10 (Reduced Inequalities) by promoting equitable access to essential services. Adult persons with musculoskeletal disabilities (MSDs) encounter many barriers to accessing Water, Sanitation, and Hygiene (WASH) services that are fundamental to health, dignity, and quality of life (2). Musculoskeletal disorders (MSDs), including conditions such as arthritis, spinal cord injuries, amputations, and congenital deformities, affect an estimated 1.71 billion people globally and represent the leading contributor to disability worldwide (3). Paradoxically, instead of effectively responding to their challenges, WASH infrastructure simply serves to exclude those with mobility impairments from access and consequently limits their health, dignity and autonomy (4). The United Nations Sustainable Development Goals (SDGs), as a whole, and SDG 6, specifically call for the sustainable management of water and sanitation, however, the inequity in accessibility particularly affects adults with MSDs in low- and middle-income countries (LMICs) where the majority of all disabled individuals live (5, 6).

The WHO/UNICEF JMP 2025 report emphasizes addressing global WASH inequalities. While safely managed drinking water reached 74% coverage by 2024, 2.1 billion people remain excluded. To support the “leave no one behind” mandate, the JMP now prioritizes disaggregating SDG data by disability, acknowledging that those with functional limitations face significant service disparities. This inclusive approach is further enhanced by new indicators for menstrual health and private bathing, providing a clearer view of the specific barriers to hygiene and dignity at the individual level (7).

Inadequate WASH access has severe consequences for adults with MSDs. Poorly designed facilities, such as those with narrow doorways, high steps, or lacking handrails, hinder independent use, forcing many to rely on caregivers, thereby compromising privacy and dignity (8). This inaccessibility may lead to open defecation or reduced food and water intake to avoid physical strain and embarrassment. The situation is particularly in humanitarian crises, where emergency WASH interventions often overlook disability needs. Additionally, stigma and discrimination in shared sanitation settings can cause social isolation and mental health struggles (9).

Menstrual health and hygiene management is crucial for women’s well-being, yet it remains a public health gap globally, especially in LMICs, where about 500 million women and girls lack adequate menstrual management facilities (10). Women with disabilities face compounded challenges due to limited healthcare access, mobility restrictions, and caregiver dependence, exacerbating menstrual health management (MHM) disparities (11). Research often overlooks disability-specific issues, focusing instead on general population practices like cloth reuse.

Research on the intersection of disability and WASH insecurity, particularly for those with MSDs, is limited. While some studies address general WASH barriers for people with disabilities, few focus specifically on adults with MSDs, whose mobility issues demand tailored solutions (12). Evidence indicates that simple modifications, such as installing grab bars, constructing ramps, and providing ground-level toilets, can greatly enhance accessibility (13). However, WASH programs often lack inclusive design, and policymakers seldom consult people with disabilities during planning, perpetuating neglect and exclusion (4). To understand these multilevel influences, this review applies the Socio-Ecological Model as an organizing framework to synthesize evidence across individual, interpersonal, community, institutional, and policy levels.

This systematic review aims to address these gaps by synthesizing evidence on WASH-related challenges faced by adults with MSDs. The objectives are to: (1) identify barriers limiting WASH access and use among adults with MSDs; (2) explore facilitators that improve access; and (3) summarize available interventions. By evaluating evidence from diverse settings, the review will provide comprehensive recommendations for inclusive WASH systems that respect the dignity and rights of adults with mobility impairments.

Ensuring accessible WASH for adults with MSDs is both a public health and human rights essential. Enhanced sanitation access can reduce infections, foster independence, and restore dignity for millions who face exclusion. As efforts toward SDG 6 continue, disability inclusion must be integral to WASH programming to fulfill the goal of “clean water and sanitation for all.”

## 2 Method

### 2.1 Study design

This systematic review aimed to identify barriers and facilitators to access and use water, sanitation, and hygiene (WASH) practices for adults with musculoskeletal disabilities, as well as report any interventions that have been undertaken. The review process followed the Preferred Reporting Items for Systematic Reviews and Meta-Analyses (PRISMA) 2020 guidelines for a systematic and transparent process. This review was reported in accordance with the PRISMA 2020 guidelines. This review was designed as a qualitative evidence synthesis, and therefore included qualitative studies only to enable an in-depth exploration of barriers, facilitators, and contextual factors.

### 2.2 Eligibility criteria

The inclusion and exclusion criteria were established in alignment with the research objectives.

Inclusion Criteria

- Peer-reviewed and primary research articles.
- Studies involving adults (≥18 years) with musculoskeletal disabilities.
- Studies exploring barriers, facilitators, or interventions related to WASH.
- Qualitative or mixed-method study designs (only qualitative findings were extracted from mixed-method studies).
- Full-text articles available in English.
- No restriction was applied on the year of publication.

Exclusion Criteria

- Studies involving children only.
- Studies that were not primary research.
- Conference abstracts, proceedings, and non–peer-reviewed publications were excluded.

### 2.3 Information sources and search strategy

A comprehensive and systematic search was performed across multiple electronic databases, including MEDLINE, Embase, and CENTRAL, to identify relevant studies. The search strategy incorporated both Medical Subject Headings (MeSH) and free-text terms, structured around four main conceptual domains: (1) musculoskeletal or physical disabilities, (2) WASH components, (3) barriers and facilitators, and (4) interventions. The search was conducted up to 01/03/2025, and no restrictions were applied regarding publication date. The detailed search strategy, including full Boolean search strings for all databases, is presented in Supplementary file 1 to ensure transparency and reproducibility.

### 2.4 Selection process

Records located through the systematic search were imported into Covidence (a systematic review management software), duplicates were removed. Two independent reviewers completed title and abstract screening to determine eligibility, excluding articles that did not meet the inclusion criteria. Full-text screening was conducted on the remaining articles to assess relevance, with reasons for exclusions reported as PRISMA guidelines. Eligible studies were included for analysis.

To ensure the review specifically addressed musculoskeletal disabilities (MSDs), a targeted extraction strategy was applied to mixed-disability studies. Data were included only if they provided disaggregated results for mobility impairments or identified barriers directly impacting the functional limitations of MSDs. This manual screening allowed for the isolation of relevant evidence from broader disability research.

### 2.5 Data collection process

Data extraction was performed using a standardized form within Covidence, capturing study characteristics such as author(s), publication year, country, objectives, study design, participant details, and type of musculoskeletal disability. Information on WASH-related barriers, facilitators, and interventions were extracted, including key findings. Additional variables included study characteristics, population demographics, and qualitative outcomes were documented.

### 2.6 Quality assessment

The methodological quality of included studies was assessed using the NIH Quality Assessment Tool. This tool, developed by the National Heart, Lung, and Blood Institute (NHLBI) in 2013, focuses on potential flaws related to study design, implementation, and data collection. Each study included was assessed independently, and the final quality ratings (good, fair, or poor) were recorded. Two reviewers independently evaluated each study across key criteria, rating them as good, fair, or poor. Disagreements were resolved through discussion. Ratings were used to inform interpretation but not to exclude studies.

### 2.7 Synthesis methods

The key findings from each study were coded line-by-line, and assigned into descriptive themes regarding barriers, facilitators, and interventions. These descriptive themes were developed into analytical themes, to identify patterns and contextual understanding. The synthesis was undertaken manually, using Microsoft Word and Excel, and in the absence of qualitative data analysis software. The identified barriers and facilitators were grouped into themes and subsequently mapped onto the Socio-Ecological Model (individual, interpersonal, community, institutional, and policy levels) to construct the conceptual framework. Potential biases were minimized by systematic data extraction and employing independent review.

The assignment of barriers and facilitators to specific SEM levels was conducted deductively, based on the primary level at which the influence operated. Knowledge-related factors were mapped to the individual level; stigma and social support to interpersonal or community levels; infrastructure-related barriers to the institutional level; and governance or planning issues to the policy level.

## 3 Results

### 3.1 Study selection

**Figure 1:**
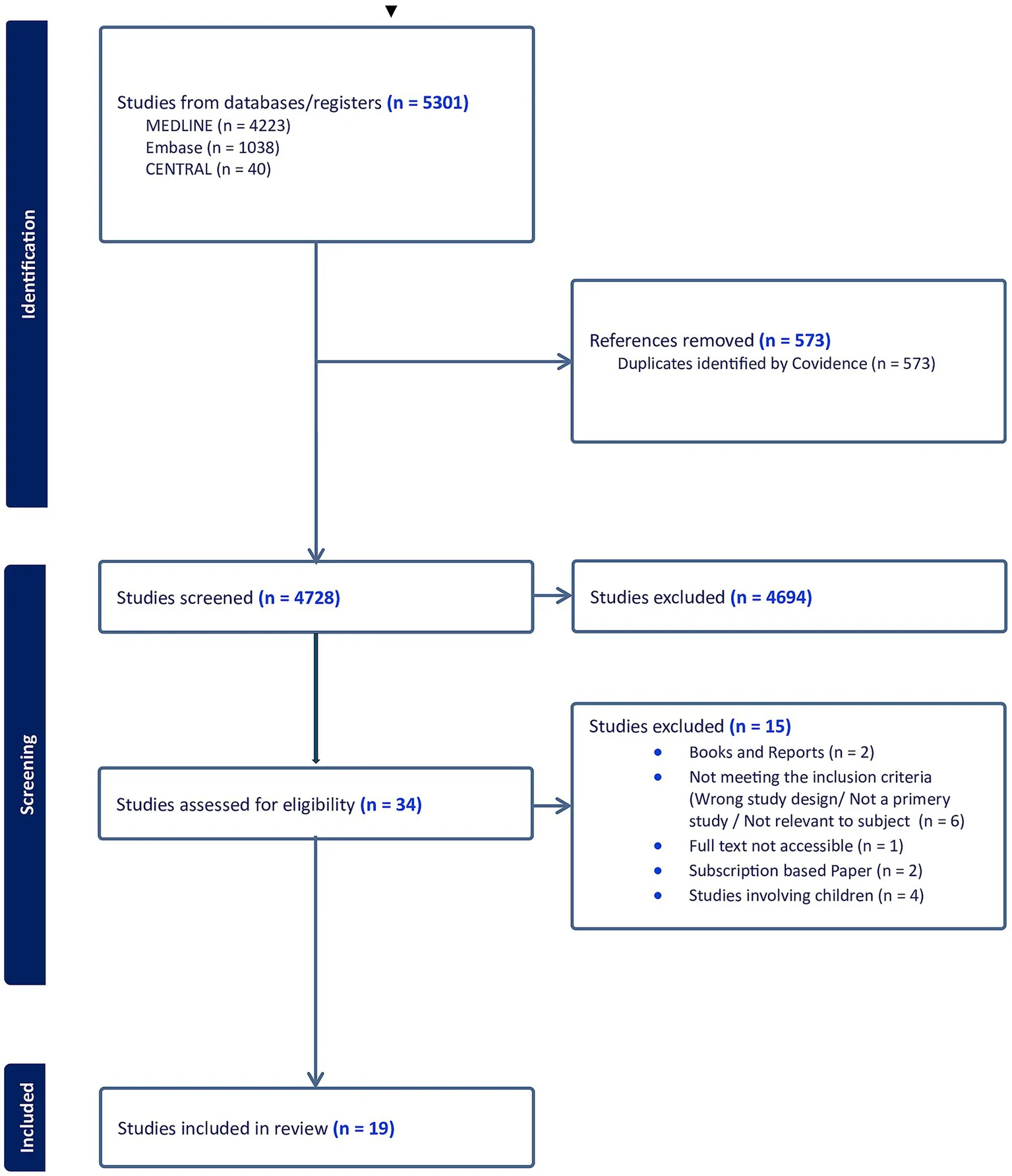
Preferred reporting items for systematic reviews and meta-analyses.

A total of 5,301 references were identified and imported into Covidence for screening. After removing 573 duplicates, 4,728 unique studies remained. Screening titles and abstracts led to the exclusion of 4,694 studies. Full-text assessment was conducted for 34 studies, of which 15 were excluded due to reasons such as not being primary studies, involving children, subscription requirements, or irrelevant content. Ultimately, 19 studies were identified as fulfilling the inclusion criteria and therefore could be included in the review. The Preferred Reporting Items for Systematic Reviews and Meta-Analyses (PRISMA) flow diagram is illustrated in Figure 1. Table 1 provides detail of the characteristics of the studies that were included with a summary of the study design, country of sample, population, type of intervention implemented, and the outcome measures used to measure effectiveness.

**Table 1:** Summary of study characteristics, interventions, and outcome measures of included studies.

Authors id	Country	Objective	Study design	Population	Intervention/exposure	Outcome measure	Quality appraisal ratings
Mohamed 2022 (19)	Tanzania	To evaluate WASH access for people with disabilities, COVID-19 measures, and existing challenges.	Cross-sectional qualitative study.	102 participants with disabilities	NA/Observational study	Challenges in accessing WASH and adhering to COVID-19 preventive measures	Good
Khan 2024 (20)	Bangladesh	To assess menstrual hygiene practices and challenges among women with disabilities	Cross-sectional study	51,535 women with disability	NA/Observational study	Appropriate material uses for menstrual hygiene and access to private washing/changing space with Impact of menstruation on social activities, school, or work	Fair
Davies 2024 (21)	Malawi	To assess the level of participation of individuals with disabilities in a Community-Led Total Sanitation (CLTS) intervention and identify barriers to their inclusion.	Cross-sectional study	247 households and 281 individuals with disability	CLTS intervention	Lower participation of disabled individuals in key activities	Fair
Wilbur 2022 (16)	Vanuatu	To assess menstrual hygiene challenges and social participation among individuals with disabilities	Mixed-methods, population-based study	Case–control study: 164 menstruators with disabilities and 169 without disabilities Qualitative sample: 20 menstruators (12 with disabilities and 8 without).	Focus on inclusive water, sanitation, and hygiene (WASH) policy.	Privacy and safety during menstruation and impacts on social participation.	Good
Mactaggart 2021 (22)	Vanuatu	To assess disability prevalence and explore WASH barriers, stigma, and caregiver reliance, especially for those managing menstruation or incontinence.	Case–control study	814 people with disabilities and 702 without disabilities	Inclusive WASH interventions focusing on stigma reduction and menstrual / incontinence products	Disability prevalence: 2.6%; rural vs. urban WASH access and barriers to WASH for people with disabilities vs. those without	fair
Daniel 2023 (23)	Indonesia	To assess home sanitation for PWDs and explore their knowledge, attitudes, and participation in sanitation	Mixed-method research (Quantitative surveys and Qualitative in-depth interviews and focus group discussions)	129 households surveyed; 24 in-depth interview participants.	Awareness raising on inclusive sanitation.	Inclusive sanitation access evaluated by criteria (e.g., toilet design, soap availability, privacy).	Fair
Getahun 2022 (24)	Ethiopia	To determine latrine access and identify factors associated with it among people with physically disability	Mixed cross-sectional study supplemented by qualitative methods	374 participants with physical disability	NA	Factors like wealth status and association membership and Latrine accessibility prevalence is 22%.	Fair
Wilbur 2021 (25)	Nepal	To investigate barriers to MHM that people with disabilities and their carers face	Phenomenological qualitative study	33 participants (20 individuals with disabilities and 13 carers)	NA/Observational and exploratory analysis	Barriers to MHM through qualitative outcomes such as accessibility, safety, and psychological impacts during menstruation, measured through thematic analysis.	Good
Kayoka 2019 (26)	Malawi	Post-program evaluation of an inclusive Community-Led Total Sanitation program that specifically responds to household-level needs of people with disabilities	Qualitative post-program evaluation	146 households for the audit; 12 implementers interviewed	Community-led total sanitation	Accessibility and safety of latrines	Good
Banks 2019 (27)	Nepal	To evaluate access to safe water, sanitation and hygiene (WASH), at the individual- and household-level, among people with disabilities	Case–control study	1,463 households (6,000 individuals screened) For case control (209 cases with disabilities, 209 controls)	Quality of WASH Access questionnaire	Water, sanitation, and hygiene scores and Proportions of participants facing specific difficulties (e.g., needing help with water collection, bathing)	Fair
Kuper 2018 (28)	Guatemala	To assess the Water, Sanitation and Hygiene (WASH) access and appropriateness of people with disabilities compared to those without	Nested case–control study	707 individuals with disabilities and 465 matched controls	NA/Observational study	Difficulties in sanitation and hygiene practices (e.g., using toilets or bathing) and associated barriers.	Fair
Mactaggart 2018 (29)	Bangladesh, Cameroon, India, and Malawi	To assess access to adequate water, sanitation and hygiene (WASH) among people with disabilities at the household and individual level	Cross-sectional survey	99,252 participants across datasets; 2,494 with disabilities	Cross-sectional surveys	Disability prevalence estimates and Comparison of improved vs. unimproved sanitation and Access vs. no access to WASH facilities	Fair
Geere 2018 (30)	South Africa, Ghana, and Vietnam	To find the impact of water carrying on spinal health and its role in the musculoskeletal disease burden	Cross-sectional survey	A total of 3,365 people with disabilities across the three countries	N/A (Observational study)	Self-reported general health, pain locations, and disability.	Fair
Biran 2018 (31)	Malawi	To evaluate an intervention to improve inclusion of people with disability in CLTS through training facilitators	Mixed-methods: Quantitative (cluster-randomized trial) and qualitative (in-depth interviews)	171 people with disabilities (78 in control, 93 in intervention).	Inclusive CLTS, awareness training for facilitators and community	Proportion of households improving latrine access for people with disabilities	Fair
White 2016 (9)	Malawi	To explores the WASH priorities of disabled people and uses the social model of disability	Qualitative study using thematic analysis of various participatory methods (interviews, photo voice, emotion mapping).	51 (36 disabled individuals and 15 carers).	No direct intervention	Barriers and needs identified during WASH practices	Good
MacLeod 2014 (32)	Cambodia	To assess challenges in water and hygiene access for people with physical disabilities and inform policy for rural water and sanitation interventions.	Qualitative study (semi-structured interviews, thematic analysis)	15 respondents with disability	Household water treatment systems (HWTS), such as biosand filters (BSFs)	Access to Water, Sanitation and Hygiene Services and Other Preventive Measures against COVID-19 among People with Disabilities	Fair
Nowreen 2022 (33)	Bangladesh	SWOT for water and sanitation access to hygiene and MHM practices from the gender perspectives	1. Mixed-method approach: 2. Focus group discussions (FGDs). 3. In-depth interviews (IDIs). 4. Key informant interviews (KIIs). SWOT analysis (Strengths, Weaknesses, Opportunities, Threats	15 disabled females: 10 in Korail, 5 in Kalyanpur 30 IDIs (15 disabled females, 15 non-disabled females). 22 key informants.	Disabled-friendly toilet facilities, Vocational training for disabled females and Affordable menstrual hygiene products	SWOT outcomes measured through qualitative data from FGDs, IDIs, and KIIs at a single time point.	Good
Hey 2022 (34)	Uganda	To develop and evaluate a sustainable solution to improve sanitation access in rural areas	Qualitative research and product development evaluation	Approximately 20 interviews conducted with disabled individuals	Implementation of three types of portable pit latrine assistive devices	Device usability and acceptance evaluated through field testing over multiple years	Fair
BerhanuAsfaw 2016 (35)	Ethiopia	To determine latrine access and utilization, and explore the challenges in latrine use among PPD (people with physical disabilities)	Cross-sectional study	419 participants with physical disability	No direct intervention	Factors like family support and latrine accessibility	Fair

### 3.2 Quality assessment

The risk of bias assessment was conducted using the NIH Quality Assessment Tool, which evaluates the internal validity of studies by examining critical methodological aspects. Of the 19 included studies, 6 were rated as good and 13 as fair using the NIH Quality Assessment Tool. The good-quality studies demonstrated clear objectives, appropriate designs, and reliable outcome measures. The fair-quality studies had minor methodological concerns, such as limited reporting on confounders or sampling strategies. No studies were rated as poor or excluded based on quality, as the limitations did not significantly affect the synthesis. Full quality ratings are available in Supplementary file 2.

Table 2 summarizes the characteristics of participants across the 19 included studies. Participants ranged from 18 years and above in most studies, although some included broader age ranges. Both males and females were represented. While several studies included multiple disability types, this review extracted and analyzed findings specific to adults with musculoskeletal (physical) disabilities only. Physical impairments were the primary focus of the included evidence. Most studies were conducted in rural or mixed rural–urban settings. Severity of disability was generally not reported.

**Table 2:** Participant characteristics of included studies.

Authors id	Age (mean/range)	Sex (M/F)	Disability type and severity	Rural/Urban
Mohamed 2022 (19)	24–77	56/46	Physical (34), Albinism (27), Hearing impairment (22); Severity: not reported	Rural (majority)
Khan 2024 (20)	15–49	Women only	Vision (12.2%), Cognitive (9.5%), Walking (8.2%); Severity: not reported	Both rural & urban
Davies 2024 (21)	18–65	49/232	Mobility (85%), Self-care (38%), Hearing (5%), Vision (4%), Cognition (0%); Severity: not reported	Rural
Wilbur 2022 (16)	18–45	Only girls (menstruators)	Seeing, Hearing, Mobility, Cognition (disability vs. non-disabled); Severity: not reported	Both rural & urban
Mactaggart 2021 (22)	18+	24,808/23,668	Seeing, Hearing, Walking, Self-care, Understanding; Severity: not reported	Both rural & urban
Daniel 2023 (23)	Mean = 44.8	32/97	Physical hand/foot (33.3%), Mute (27.1%), Paralyzed (24.8%), Blind (19.4%), Deaf (7.8%); Severity: not reported	Rural
Getahun 2022 (24)	Mean 44.8	177/197	Physical disability; Severity: not reported	Not reported
Wilbur 2021 (25)	15–24	0/20	Mobility (8), Visual (3), Hearing (2), Cognition (NR); Severity: not reported	Urban (16), Rural (4)
Kayoka 2019 (26)	18+	6/4	Physical, Visual, Hearing, Cognitive; Severity: not reported	Rural
Banks 2019 (27)	15–75	208/173	Sensory (hearing/seeing): 36%, Physical (mobility/upper body/fine dexterity): NR; Severity: not reported	Both rural & urban
Kuper 2018 (28)	≥18	253/454	Seeing (28%), Hearing (15%), Physical (31%), Anxiety/Depression (44%), Self-care (11%); Severity: not reported	Both rural & urban
Mactaggart 2018 (29)	0–65	45,333/53,921	Hearing, Visual, Physical, Intellectual; Severity: not reported	Both rural & urban
Geere 2018 (30)	Mean 22.2	1,572/1,793	Physical disability; Severity: not reported	Both rural & urban
Biran 2018 (31)	10–70	87/84	Hearing, Seeing, Physical disability; Severity: not reported	Rural & peri-urban
White 2016 (9)	Mean 36	20/16	Physical (55), Visual (8), Hearing (5), Cognitive/Intellectual (15), Other (epilepsy/blood disorder): 5; Severity: not reported	Both rural & urban
MacLeod 2014 (32)	≥18	15/0	Physical disability; Severity: not reported	Rural
Nowreen 2022 (33)	12–30	0/15 disabled females	Physical disability; Severity: not reported	Rural (both sites: Korail, Kalyanpur – informal settlements)
Hey 2022 (34)	18+	Not reported	Physical disability; Severity: not reported	Rural
BerhanuAsfaw 2016 (35)	Mean 29.9	229/190	Physical disability; Severity: not reported	Rural

Table 3 presents a summary of the key findings, highlighting the main barriers and facilitators affecting access to and use of WASH services among adults with disabilities. The findings from the included studies were synthesized and organized into seven thematic domains: physical environment, socioeconomic factors, social/cultural barriers, gender-specific barriers, information and awareness, workplace barriers, and policy/system-level factors. Within each domain, barriers and facilitators were identified and summarized.

**Table 3:** Summary table of key findings (barriers and facilitators).

Domain	Barriers	Facilitators
Physical environment	Lack of ramps, handrails, step-free access Narrow doorways, inaccessible latrines Distance to water source	Structural modifications (ramps, handrails, wider doors) Accessible toilet designs and home adaptations
Socioeconomic factors	Poverty and unemployment limit ability to adapt WASH infrastructure	Affordable WASH solutions (e.g., biosand filters) Community-level low-cost innovations
Social/cultural barriers	Stigma, exclusion, and shame (esp. menstrual hygiene) Poor participation in CLTS and WASH programs	Family/caregiver support (e.g., help with water collection, hygiene) Community engagement and male family help
Gender-specific barriers	Inadequate menstrual hygiene infrastructure and supplies Lack of privacy and safety	Inclusive MHM education Gender-sensitive infrastructure (e.g., private toilets with MHM supplies)
Information & awareness	Limited knowledge on accessible WASH designs Poor communication about services and emergency WASH info	Disability-inclusive communication during public health emergencies
Workplace barriers	Poor workplace WASH access compared to home	Adapted facilities at work (limited but reported)
Policy/systems	Exclusion from WASH planning and design processes CLTS models not adapted for disability inclusion	Inclusive WASH policies (e.g., training for facilitators) Active disability representation in designing policy

### 3.3 Interventions reported

The included studies reported various interventions to improve WASH access for people with disabilities, ranging from low-cost adaptations to policy-level changes. These included ongoing monitoring of individual WASH access, simple facility modifications like latrine chairs and handrails, and promoting inclusive participation in programs like CLTS, though participation and information access remain limited. Economic constraints highlighted the need for affordable solutions such as biosand filters. Menstrual hygiene management (MHM) required accessible facilities, caregiver support, and stigma reduction. During COVID-19, communication gaps exposed the need for inclusive messaging. Infrastructure recommendations included accessible toilets, ramps, lighting, piped water, and menstrual supplies for disabled women. Standardizing inclusion in large-scale programs like Community-Led Total Sanitation (CLTS) was also emphasized.

**Figure 2:**
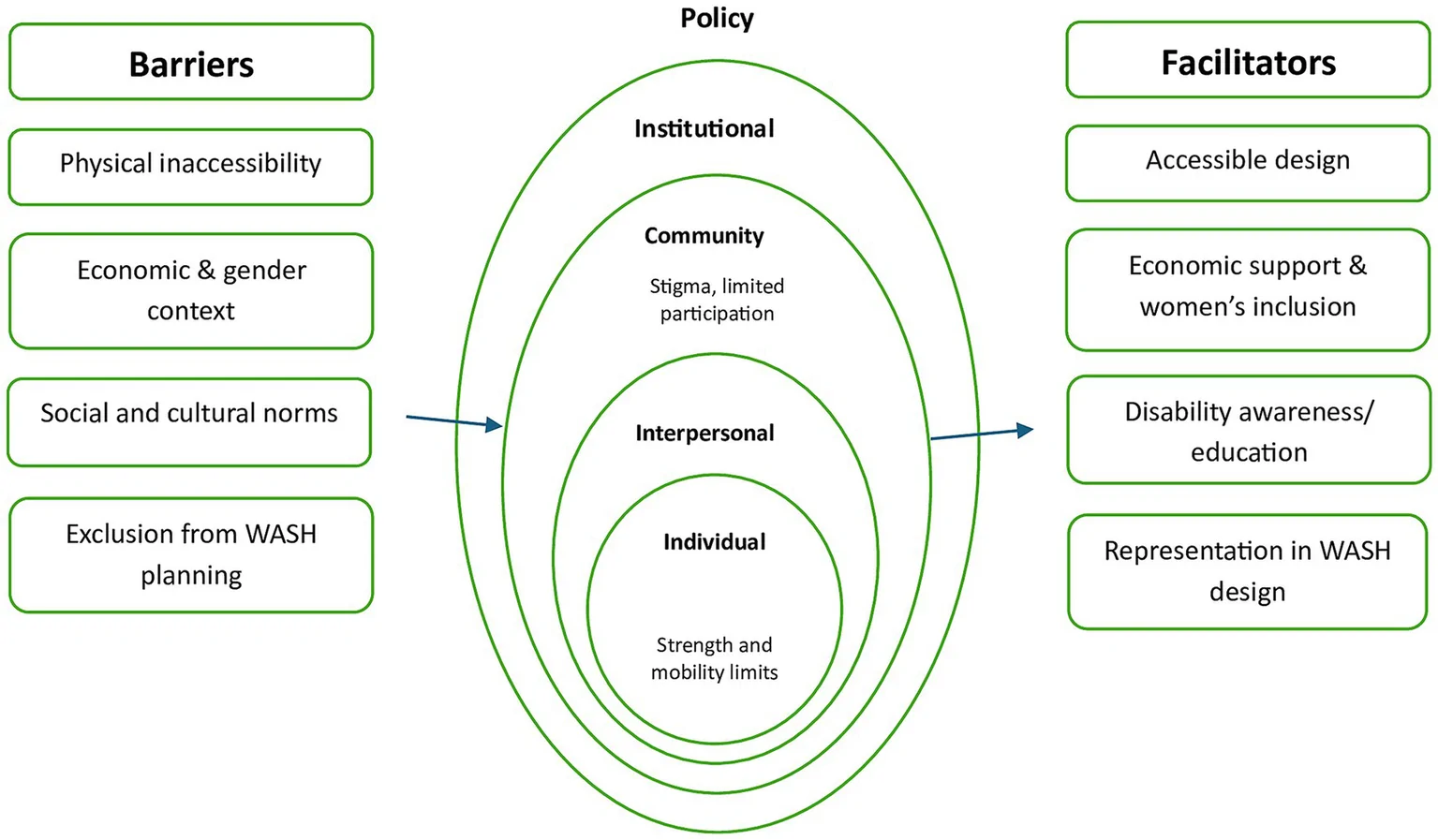
Multilevel barriers and facilitators to inclusive WASH services for people with disabilities.

Figure 2 presents the socio-ecological framework outlining the multilevel barriers and facilitators influencing access to and use of WASH (Water, Sanitation, and Hygiene) services among persons with disabilities. The framework organizes identified factors across individual, interpersonal, community, institutional, and policy levels, providing a structured conceptual overview of how influences operate at different layers of the social environment.

The framework demonstrates how barriers and facilitators interact across levels rather than functioning independently. For instance, individual mobility limitations are compounded by inaccessible physical infrastructure at the community level, while exclusion from WASH planning at institutional and policy levels reinforces structural inaccessibility. Conversely, inclusive policies and representation can positively influence community practices and household-level adaptations.

**Figure 3:**
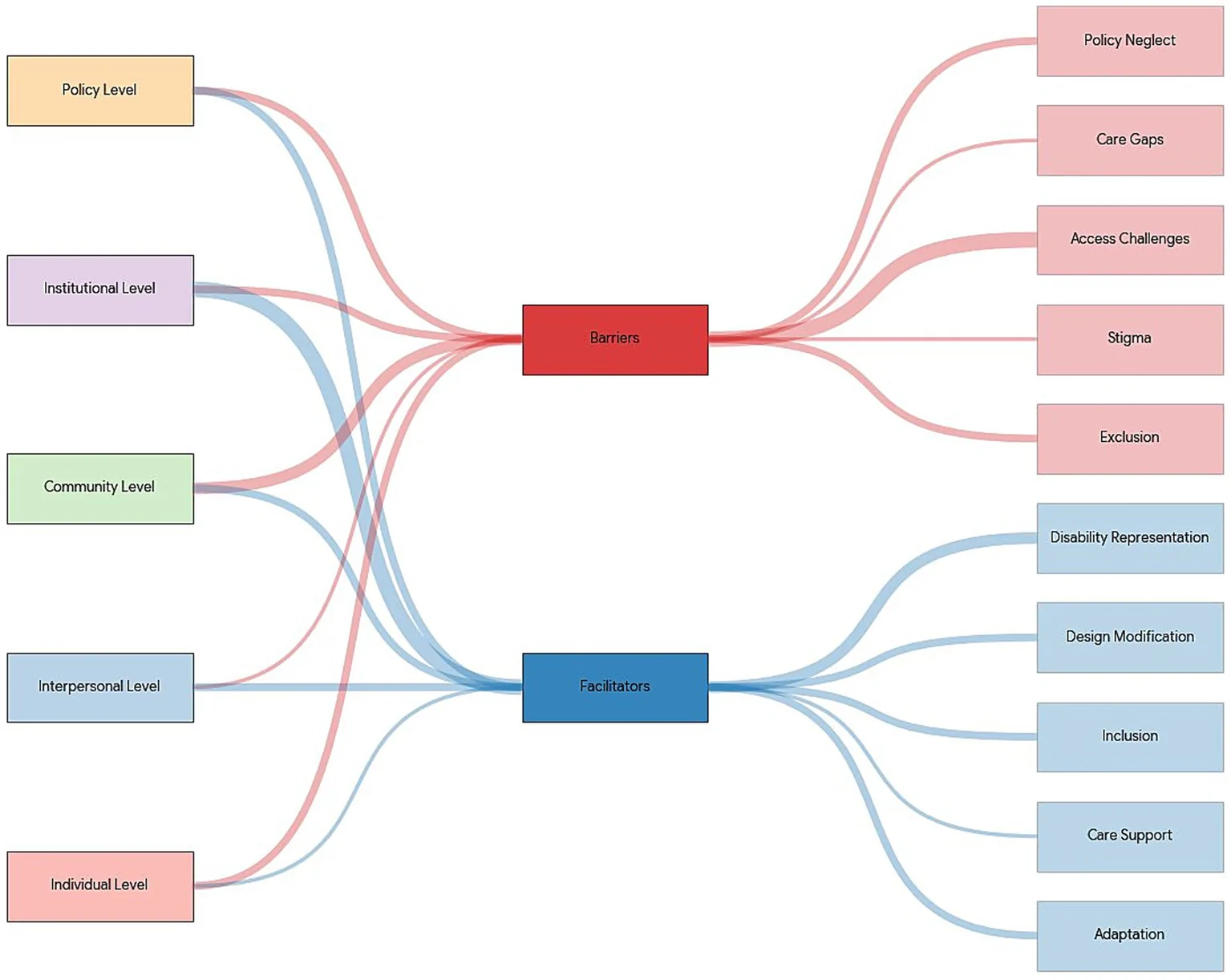
Sankey diagram showing barriers and facilitators across SEM levels influencing WASH outcomes.

Figure 3 presents a Sankey diagram that visually maps the distribution and linkages of identified barriers and facilitators across the five levels of the Socio-Ecological Model (SEM) to key WASH-related outcomes. The diagram illustrates how factors at each level connect to outcomes such as access challenges, care gaps, stigma, exclusion, adaptation, inclusion, design modifications, disability representation, and policy neglect, with color coding distinguishing barriers and facilitators.

## 4 Discussion

A total of 19 studies were included in this review, and they produced a wide range of evidence about WASH access among people with disabilities. The studies included in this systematic review identified multiple barriers and facilitators to WASH access for people with disabilities. Barriers cut across the physical, socioeconomic, social, gender-related, informational, workplace, and policy areas. Barriers included infrastructure (e.g., not physically accessible), poverty, stigma, poor attendance and participation in programs or services, lack of support for menstrual hygiene, lack of awareness, and exclusion from or poor participation in planning. Facilitators included structural changes, affordability, helpful family and community members, gender-related approaches, inclusive communications, inclusivity in establishing policies, and presence and representation involving disabilities in co-designs and policies. Reported interventions focused on low-cost modifications, inclusive education, and ‘accessibility’ needs to be focused on from the design stage and standardized in any large-scale WASH program that includes inclusion.

The results of this systematic review are consistent with the existing literature, while at the same time revealing some key differences in scope, depth, and context. Compared to previous reviews, which primarily synthesized general disability-related WASH barriers, our review specifically focused on adults with musculoskeletal disabilities and provided a structured socio-ecological synthesis of barriers and facilitators. One of the studies in low- and middle-income countries (4), in comparison their analysis primarily focused on identifying physical and social barriers that included lack of access to infrastructure, stigma, and exclusion in policy. Conversely, our review identified, specific, interventions including low-cost adaptations to infrastructure for facilities (e.g., latrine chairs, handrails), inclusive menstrual hygiene management (MHM), and low-cost options (e.g., biosand filters), and therefore addressed an important evidence gap.

Furthermore, while many emphasized a lack of policy integration and limited inclusion of disability in international WASH programs, recent global frameworks such as the UN Convention on the Rights of Persons with Disabilities (CRPD) and WHO’s 2011 World Report on Disability have since advocated more strongly for inclusive WASH policy (14, 15). However, implementation remains inconsistent. Our review supports this by revealing persistent gaps in the participation of people with disabilities in programs like Community-Led Total Sanitation (CLTS). For instance, in one study, only 7% of participants reported receiving information on accessible technologies, highlighting a significant barrier to equitable participation (16).

From a programmatic perspective, the Equity and Inclusion Framework by WaterAid provides practical advice on embedding disability inclusion into WASH programs. It confirms our findings that universal design principles and supportive caregiving greatly contribute to the participation of persons with disabilities, consistent with findings from a report by WaterAid titled Equity and Inclusion: A Rights-Based Approach (17). However, whereas some review speaks to evidence gaps and future research implications (18), our review goes further by looking at already-implemented, low-cost interventions, including latrine modifications, inclusive CLTS activities, and menstrual hygiene support, and provides useful information that could applied immediately to programs and policies.

### 4.1 Strength and limitations

This review is a rigorous and comprehensive synthesis of studies on WASH access for people with musculoskeletal disabilities among adults. A unique strength of this study is the organized categorization of barriers and facilitators in the domains of physical, social, economic, and policy-related contexts, providing detailed information for program and policy change. However, this review has limitations. First, even though the inclusion criteria stated that studies did not directly report effect sizes, a meta-analysis was not done, which limits the ability to quantify effect sizes or assess strengths of associations across the studies included. Second, although study quality was assessed using the NIH Quality Assessment Tool, studies were not excluded based on quality ratings, and findings were not weighted according to methodological rigor, which may affect the overall strength of conclusions. Additionally, only three databases (MEDLINE, Embase, and CENTRAL) were searched due to access constraints, which may have limited the identification of relevant studies. The search did not include gray literature or NGO reports, which may have resulted in omission of relevant programmatic evidence. Furthermore, only English-language studies were included, which may have introduced language bias. Additionally, limited policy evaluation studies were available, which restricts firm conclusions about the effectiveness of disability-inclusive WASH policies. Due to limited institutional access, some potentially eligible full-text articles could not be retrieved and were therefore not included, which may have introduced selection bias. Most included studies were conducted in Africa and South Asia, which may limit the generalizability of findings to other regions and contexts. Overall, despite the limitations of this review, it has provided a substantive overview of WASH practices that are disability-inclusive and called out opportunities for future research and programming.

## 5 Conclusion

This review shows that WASH policies and programs need to better include people with disabilities. Governments and organizations should work toward ensuring make sure facilities are accessible, share information in ways everyone can understand, and involve people with disabilities in decision-making. However, evidence on the effectiveness of these interventions remains limited. Future studies should test which solutions work best, particularly in relation to accessible WASH infrastructure design, disability-inclusive sanitation policy evaluation, and the cost-effectiveness of interventions, and identify ways to scale simple, low-cost strategies through community engagement and integration into existing WASH programs.

## Data Availability

The data extraction table will be available from the corresponding author upon reasonable request for academic, non-commercial use.
