# Supplementary material for: Disability and dignity: a qualitative systematic review of WASH-related barriers, facilitators and interventions for adults with musculoskeletal disabilities
Source: Front Public Health. 2026 Aug 21;14:1799013. doi: 10.3389/fpubh.2026.1799013 (PMC13541729; doi:10.3389/fpubh.2026.1799013)
Supplement: Supplementary file 1 [file Supplementary_file_1.docx]

**Information Sources and Search Strategy**

A comprehensive and systematic search was conducted across multiple electronic databases, including MEDLINE, Embase, and CENTRAL, to identify studies relevant to water, sanitation, and hygiene (WASH) practices among adults with musculoskeletal disabilities. The search included both Medical Subject Headings (MeSH**)** and free-text terms, structured around four main conceptual domains: (1) musculoskeletal or physical disabilities, (2) WASH components, (3) barriers and facilitators, and (4) interventions.

The following combination of search terms was used:

- **Disability terms**:
  "Musculoskeletal Diseases"[MeSH Terms] OR "Musculoskeletal System"[MeSH Terms] OR musculoskeletal disability OR physical disability OR mobility impairment OR musculoskeletal impairment OR "Disability, Physical"[MeSH Terms]
- **WASH-related terms**:
  "Water Supply"[MeSH Terms] OR "Sanitation"[MeSH Terms] OR "Hygiene"[MeSH Terms] OR water hygiene OR WASH OR water sanitation hygiene OR water accessibility OR hygiene practices OR sanitation access
- **Access and barrier terms**:
  barriers OR access barriers OR challenges OR obstacles OR accessibility OR facilitators OR enablers OR opportunities
- **Intervention terms**:
  interventions OR programs OR initiatives OR strategies OR "Health Programs and Plans"[MeSH Terms] OR public health interventions OR behavioral interventions OR environmental interventions

An additional simplified Boolean strategy was also used to capture broader results:
(("water" OR "sanitation" OR "hygiene") AND ("disability" OR "disabled" OR "handicap"))
